# A natural PCID2-Targeting compound suppresses hepatocellular carcinoma progression: evidence from structure-based discovery and biological evaluation

**DOI:** 10.3389/fphar.2025.1687517

**Published:** 2025-11-28

**Authors:** Zebanuer Yuemaierjiang, Jingjing Sun, Jiamin Song, Jiaping Huang, Huiyu Zhang, Lili Xi, Jingjing Guo, Xinyi Luo

**Affiliations:** 1 The First School of Clinical Medicine, Lanzhou University, Lanzhou, Gansu, China; 2 Medical Laboratory Center, The First Hospital of Lanzhou University, Lanzhou, Gansu, China; 3 Office of Institution of Drug Clinical Trial, The First Hospital of Lanzhou University, Lanzhou, Gansu, China; 4 Centre in Artificial Intelligence Driven Drug Discovery, Faculty of Applied Science, Macao Polytechnic University, Macao, China; 5 Department of Pharmacy, The First Hospital of Lanzhou University, Lanzhou, Gansu, China

**Keywords:** hepatocellular carcinoma (HCC), PCI-domain containing protein 2 (PCID2), 1,2,3,4,6-penta-O-galloyl-β-D-glucose (β-PGG), virtual screening (VS), proliferation inhibition

## Abstract

**Introduction:**

Hepatocellular carcinoma (HCC) is a highly aggressive malignancy with limited therapeutic options and poor prognosis, highlighting the urgent need for novel targets and effective agents. PCID2 (PCI-domain containing protein 2) has recently been recognized as a potential therapeutic target; however, specific inhibitors remain unidentified. Natural products, particularly monomeric compounds derived from traditional Chinese medicine (TCM), provide an important source for novel anticancer candidates.

**Methods:**

A molecular docking-based virtual screening of TCM-derived compounds were used to identify small molecules targeting PCID2. The binding interaction between the top candidate, 1,2,3,4,6-Penta-O-galloyl-β-D-glucose (β-PGG), and PCID2 was validated using surface plasmon resonance (SPR). The cytotoxicity and effects of β-PGG on HCC cell proliferation, migration, invasion, apoptosis, and cell cycle progression were evaluated *in vitro*. Exploratory analysis related to mechanisms were performed via Western blotting.

**Results:**

β-PGG was identified as a promising PCID2-targeting compound by molecular docking, and SPR confirmed its direct binding to PCID2. β-PGG significantly reduced HCC cell proliferation, migration, and invasion, while inducing apoptosis and cell cycle arrest. Treatment with β-PGG impeded the G0/G1 or S phase to G2/M phase. Mechanistically, β-PGG decreased PCID2 expression and downregulated Cyclin D1 and CDK6. At higher concentrations, β-PGG also suppressed PI3K and Akt phosphorylation.

**Discussion:**

β-PGG exhibits potent anti-HCC activity by modulating PCID2 expression, PI3K/Akt signaling, and cell cycle regulation, and it represents a promising lead compound with PCID2-targeting potential. This study not only support a rationale for further exploration of PCID2 as a therapeutic target in HCC but also provide valuable insights into the discovery of novel lead compounds from TCM for liver cancer treatment.

## 1 Introduction

Primary hepatic carcinoma (PLC) is one of the most aggressive malignancies, imposing a substantial global health burden. It ranks sixth in incidence and third in cancer-related mortality worldwide (Bray et al., 2024). The situation is equally alarming in China, where PLC is the fourth most common cancer and the second leading cause of cancer-related death (Zheng et al., 2024). Hepatocellular carcinoma (HCC), the predominant subtype of PLC, accounts for 85%–90% of cases (Wang and Deng, 2023). HCC is characterized by rapid progression, high malignancy, and poor prognosis, with a mortality rate nearly matching its incidence. The overall 5-year survival rate remains dismally low at only 18% (Storandt et al., 2022). Due to its insidious onset and aggressive nature, most patients are diagnosed at advanced or even terminal stages, precluding curative surgery. Consequently, systemic therapy has become the cornerstone of treatment for advanced HCC and, in many cases, the only therapeutic option available (Yang et al., 2023). The advent of molecular targeted therapies, exemplified by sorafenib (Llovet et al., 2008) and Lenvatinib (Kudo et al., 2018), has marked a transformative era in HCC treatment. More recently, immune checkpoint inhibitors (ICIs) targeting PD-1/PD-L1 have further expanded treatment options. However, responses remain heterogeneous due to the complexity of the tumor immune microenvironment (TIME), prompting the development of innovative strategies such as radiomics-based immune profiling to improve patient stratification and therapeutic precision (Wu et al., 2023). In parallel, emerging studies have highlighted that targeting immunosuppressive signaling, for example, by modulating the glucocorticoid receptor (GR) pathway, can reshape the TIME, increase CD8^+^ T cell infiltration, and enhance the efficacy of tyrosine kinase inhibitors including Lenvatinib (Wang et al., 2024). Nevertheless, despite these advances and increasingly diversified treatment options, the long-term prognosis of HCC remains unsatisfactory, with only modest improvements in survival outcomes (Forner et al., 2018). This emphasizes the urgent need to develop novel therapeutic strategies. Natural small-molecule inhibitors represent a promising avenue, particularly those targeting key regulators of the cell cycle, as they may improve both prognosis and therapeutic efficacy (Otto and Sicinski, 2017). Identifying specific molecular targets involved in cell cycle regulation and discovering corresponding lead compounds could pave the way for more effective therapeutic strategies against HCC.

PCI domain-containing protein 2 (PCID2), located on chromosome 13q34 and known as CSN12-like protein, is structurally homologous to yeast Thp1. As a core component of the transcription and export complex 2 (TREX2) complex, PCID2 plays a crucial role in mRNA export from the nucleus to the cytoplasm. Additionally, it regulates cell cycle checkpoints, prevents R-loops formation, and maintains protein stability, and modulates the transcription of lymphoid lineage-specific genes (Crespo et al., 2024; García-Muse and Aguilera, 2019; Vdovina et al., 2023). Our previously work identified a cinnamamide derivative, FO (N-*trans*-feruloyloctopamine), isolated from *garlic peel*, which exhibited strong inhibitory effects on HCC cell proliferation and invasion (Bai et al., 2017). Mechanistic studies further revealed that PCID2 is frequently over-expressed in HCC tissues and correlates with tumor progression and poor prognosis (Bai et al., 2022). Elevated PCID2 expression enhances malignancy, invasiveness, and metastatic potential, implicating PCID2 as a driver of cell cycle dysregulation and apoptosis resistance, thereby promoting tumor proliferation and invasion. Additionally, PCID2 dysregulation has been implicated in several other malignancies, including colorectal cancer, gastric cancer, and breast cancer (Gondo et al., 2021; Jie et al., 2024; Zhang et al., 2021). Collectively, these findings highlight PCID2 as a critical regulator of tumor initiation and progression.

Given this evidence, PCID2 emerges as a novel and potential therapeutic target for HCC drug development. However, research on PCID2 inhibitors remains at an early stage, and no molecules specifically targeting PCID2 have yet been reported. This may be attributable to the incomplete elucidation of PCID2’s biological functions and/or the lack of efficient screening methodologies. Virtual screening (VS), a widely applied high-throughput screening approach and an extension of artificial intelligence (AI)-driven drug design, provides a powerful framework for drug discovery. By guiding the identification of drug candidates and lead compounds, VS substantially reduce the randomness and inefficiency traditional screening methods. Meanwhile, increasing interest in traditional Chinese medicine has emphasized the therapeutic potential of natural compounds, which often exhibit low toxicity and fewer side effects, making them attractive candidates for developing tumor-specific inhibitors (Luo et al., 2019).

**FIGURE 1:**
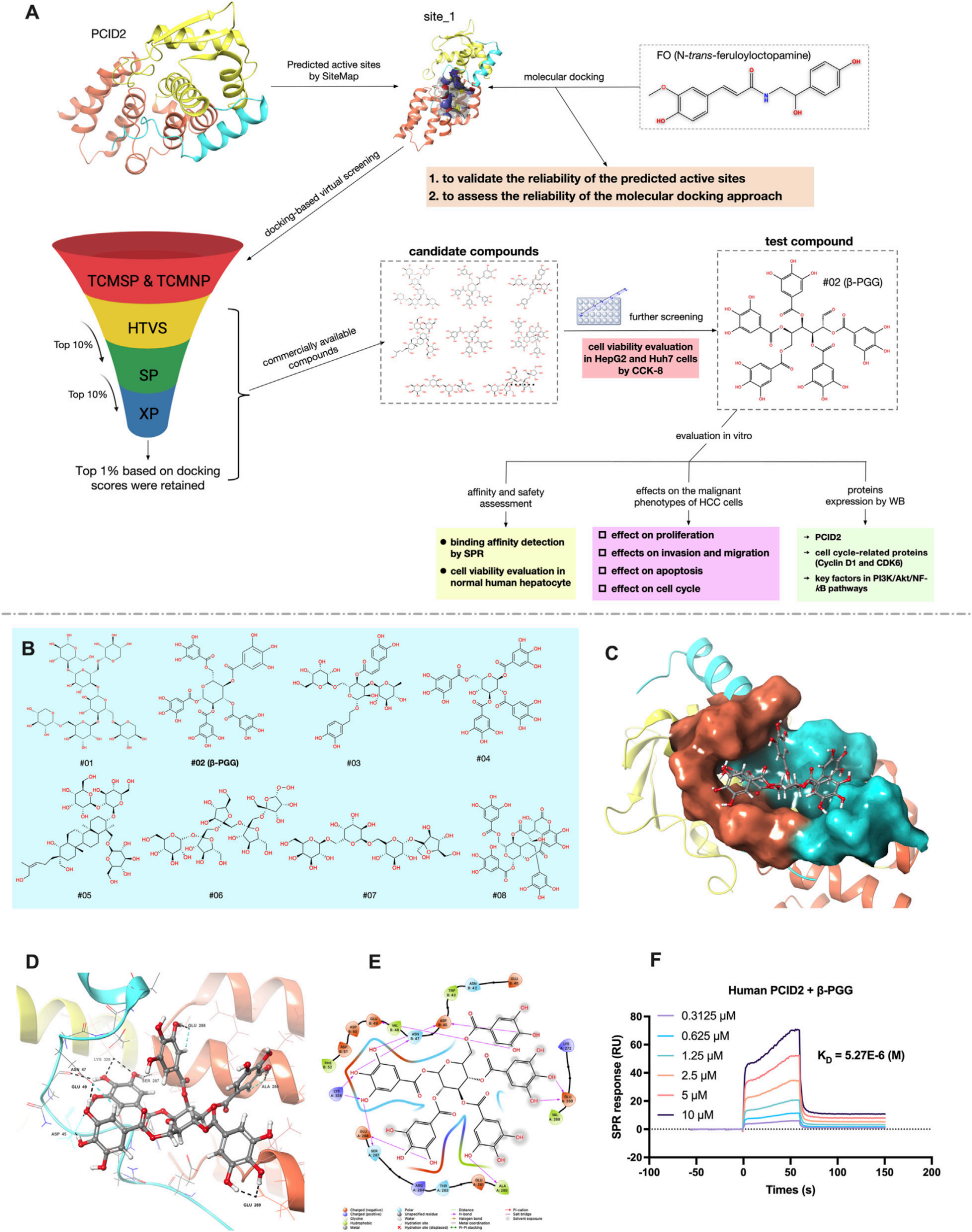
**(A)** Schematic diagram of the screening strategy for potential PCID2 inhibitors and activity evaluation workflow in this study. **(B)** The 2D structures of eight candidate compounds. **(C)** Surface representation of the compound #02 docked pose at the active site_1 of PCID2. Orange represents the superhelical domain of PCID2, and cyan represents the domain of DSS1. **(D,E)** Binding mode of the compound #02 in active sites (residues within 3 Å and 2D-interaction was shown). **(F)** The binding affinity between human PCID2 and varying concentrations of the compound #02 (β-PGG) was assessed by SPR, and the K_D_ value were calculated.

In this study, a hierarchical VS strategy based on molecular docking was utilized to identify potential natural PCID2 inhibitors. Eight candidate compounds were selected for *in vitro* evaluation of cell viability using the CCK-8 assay. Among them, one hit compound, 1,2,3,4,6-Penta-O-galloyl-β-D-glucose (β-PGG), was chosen to investigate its effects on key malignant phenotype of HCC cells, including proliferation, invasion, migration and apoptosis. Its pharmacological activity against HCC was validated at the cellular level. Surface plasmon resonance (SPR) was further applied to evaluate the binding affinity between β-PGG and PCID2, and the impact of β-PGG on the viability of normal human hepatocytes was also assessed. Furthermore, we explored the underlying mechanism by which β-PGG suppresses HCC cells proliferation through PCID2 inhibition. Figure 1A illustrates the screening strategy and activity evaluation workflow employed in this study. Collectively, this strategy establishes a rapid and effective strategy to discovering novel PCID2 inhibitors from natural sources. More importantly, our findings provide new mechanistic insights and highlight PCID2-targeted inhibition as a promising therapeutic avenue for HCC treatment.

## 2 Materials and methods

### 2.1 Protein preparation, active sites prediction and grid generation

The 3D structure of human PCID2 was retrieved from Protein Data Bank (PDB code: 3T5X) (Ellisdon et al., 2012) and prepared using the Protein Preparation Wizard (Epik, 2021; Impact, 2021; Prime, 2021) in Schrödinger software. This involved adding hydrogens, assigning charges using OPLS_2005 force field, protonation states at pH 7 ± 2, and energy minimization using Impact Refinement module.

The active sites of the processed protein structure were analyzed using the SiteMap module (SiteMap, 2021). A minimum of 15 probes were selected to cover potential binding pockets. The probe radius was set to the default value of 1.0 Å, the grid resolution was defined as “Standard” and the option “Exclude water molecules” was applied to minimize interference from solvent molecules. All other parameters were default.

Receptor Grid Generation module (Glide, 2021) was used to calculate the binding site based on the information obtained from the SiteMap analysis, and generate the grid center coordinates, while the grid box size was maintained at the default parameter settings. The generated grid files were used for docking in Glide program.

### 2.2 Molecular docking, molecular dynamics simulations and binding free energy calculation

To evaluate the predicted active site and assess the reliability of the docking protocol, FO, previously identified by our group as a PCID2 inhibitor (Bai et al., 2017), was selected as a reference ligand and docked into PCID2 using the Glide module (Glide, 2021) with a van der Waals radii scaling of 0.8 Å and partial charge cutoff of 0.15.

Molecular dynamics (MD) simulations were performed using the AMBER 18 package (Cerutti et al., 2018) on two systems: apo-PCID2 and FO-PCID2 complex. Protein parameters were described by the AMBER ff14SB force fields (Maier et al., 2015). Each system was solvated in an approximately 10 × 10 × 10 Å^3^ rectangular box using the TIP3P water model (Jorgensen et al., 1983). Na^+^ and Cl^−^ ions were added to the hydrated system to ensure system neutrality.

The MD simulations protocol consisted of energy minimization, heating, equilibration, and production. Initial energy minimizations (four stages of 5,000 steps each) were performed using the steepest descent followed by conjugate gradient algorithm. Systems were then heated from 0 to 310 K over 50 ps. Equilibration was conducted in the NPT ensembles with gradually decreasing force constants of 5.0, 2.5, 1.0, 0.5, 0 kcal/mol/Å^2^ applied to non-hydrogen atoms and heavy atoms of proteins for 200 ps each. Production runs were performed for 200 ns under NPT conditions without restraints. Long-range electrostatic interactions were calculated using the Particle Mesh Ewald (PME) method (Essmann et al., 1995) with a 10 nm cutoff, and bond vibrations involving hydrogen were constrained using SHAKE algorithm (Ryckaert et al., 1977). Trajectories were recorded every 2 fs, and analyses were performed using CPPTRAJ (Roe and Cheatham, 2013). Three independent replicates were simulated for each system.

Equilibrated trajectories were analyzed by clustering and principal component analysis to identify the most representative conformations for evaluating ligand-receptor interactions, including hydrogen bonding, electrostatic, and hydrophobic interactions. The binding free energy between PCID2 and FO was estimated using the MM-GBSA method, and per-residue energy decomposition was conducted to identify the residues contributing most significantly to binding.

### 2.3 Compounds dataset: source and preparation

In a total of 121341 compounds retrieved from Traditional Chinese Medicine Systems Pharmacology Database (TCMSP) (Ru et al., 2014), and Traditional Chinese Medicine Natural Product library (TCMNP) (Chen, 2011) were used for virtual screening in this work. Energy minimization was performed by Impact module, using OPLS_2005 force field with a distance-dependent dielectric and conjugate gradient algorithm, and others were default values. All the optimized compounds were prepared with LigPrep module (LigPrep, 2021) before docking, and all species existing at pH = 7 ± 2, including tautomers and enantiomers, were generated. Using OPLS_2005 force field in vacuum, a short conformational search was performed to relax the structures.

### 2.4 Molecular docking-based virtual screening

A hierarchical docking strategy was implemented to screen preprocessed compounds into the binding pocket of PCID2 using the Glide Ligand Docking module. Primary filtering was performed via high-throughput virtual screening (HTVS), and the top 10% of compounds ranked by docking scores were advanced to secondary evaluation. Standard precision (SP) docking was then applied, again retaining the top 10% of compounds. Finally, extra precision (XP) docking was conducted to achieve high-accuracy predictions, with the top 1% of scoring compounds selected as high-potential candidates. All docking steps used default parameters without additional spatial constraints, and for each compound, the five lowest-energy conformational isomers were automatically saved.

### 2.5 Surface plasmon resonance (SPR)

The SPR technique, which is a robust method for analyzing real-time biomolecular interactions without the need for labeling, was utilized to identify the interaction affinity between the human PCID2 protein and the selected compound #02 (i.e., β-PGG) The PCID2 protein was directly coupled to the CM5 Sensor chip (BR-1005–30, Cytiva) for determination. Following incubation, a gradient of β-PGG was introduced into the PCID2 protein-CM5 system. The dissociation constant (K_D_), association rate constant (K_a_) and dissociation rate constants (K_d_) were obtained by globally fitting the data to a 1:1 Langmuir binding model using the Biacore Insight evaluation software (Cytiva, Marlborough, MA, USA).

### 2.6 Cell lines

The human hepatocellular carcinoma HepG2 cell line was obtained from the Institute of Basic Medical Sciences, Chinese Academy of Medical Sciences, while Huh7 cells were purchased from Zhongqiao Xinzhou Biotechnology Co., Ltd.

### 2.7 Chemical and reagents

β-PGG (CAS No.: 14937–32-7; HPLC ≥99%) was purchased from Shanghai Yuan Ye Company; MEM medium (Batch No. SH30024.01). DMEM medium (Batch No. SH30022.01), and 0.25% trypsin-EDTA solution (Batch no: SH30042.01) were obtained from Hyclone. Penicillin-streptomycin double antibody solution (Batch No.: SV30010) was purchased from Cytiva, while CCK-8 assay kit (Batch No.: K-1018) was sourced from APExBIO. Matrigel matrix gel (Batch No.: 356231) was obtained from Corning. 4% paraformaldehyde solution (Batch No.: PMK0240) was purchased from Pumi Biological Co., Ltd. Amine oxalate crystalline violet staining solution (Batch No.: G1063-100 ML) was obtained from Beijing Solepol. the Annexin V-FITC apoptosis detection kit (Batch No.: E-CK-A211-50Assays) and cell cycle detection kit (Batch No.: E-CK-A351) were purchased from Elite Bioscience & Technology Co., Ltd.

For protein extraction and Western blotting, RIPA lysis buffer (Batch No.: R0010) was obtained from Solepol Biotechnology Co., Ltd., while the BCA protein quantification kit (Batch No.: 23227) was procured from Thermo Fisher Scientific. The 10% ExpressCast PAGE Color Gel Rapid Kit (Batch No.: P2012) was supplied by New Saimae Biotechnology Co., Ltd. Antibody dilution buffer (Batch No.: SW162-01) and rapid blocking solution (Batch No.: SW161-01) were purchased from Severn Biotechnology Co., Ltd. Horseradish peroxidase (HRP)-conjugated goat anti-rabbit and anti-mouse secondary antibodies (Batch No.: PMK-014–090M/PMK-014-091) were obtained from Wuhan Pumic Biotechnology Co., Ltd. Primary antibodies against GAPDH (Batch No.: 60004-1-IG) and β-actin (Batch No.: 20536-1-AP) were sourced from Beijing Yunpeptide Biotechnology Co., Ltd. PCID2 (Batch No.: GTX52023) was procured from GeneTex, while CDK6 (Batch No.: ab124821) and CyclinD1 (Batch No.: ab16663) were obtained from Abcam UK. Antibodies against *p*-PI3K (Batch No.: 4228T), PI3K (Batch No.: 4292S), *p*-Akt (Batch No.: 4060T) and Akt (Batch No.: 4691T) were purchased from Cell Signaling Technology, USA. The polyvinylidene difluoride (PVDF) membrane (Batch No.: IPVH00010) was obtained from Millipore, USA, and the chemiluminescence detection kit (Batch No.: 180–5,001) was purchased from Shanghai Tianneng Technology Co., Ltd.

### 2.8 Cell culture

HepG2 cells were cultured in MEM complete medium, and Huh7 cells were maintained in DMEM medium, both supplemented with 10% fetal bovine serum (FBS) and 1% penicillin-streptomycin. Cells were incubated at 37 °C under 5% CO_2_ with saturated humidity, and when the cell density grew to more than 80%, the cells were sub-cultured at a 1:2 ratio for subsequent experiments.

### 2.9 Construction, transfection, and lentivirus infection

The HepG2 cell was seeded in 6-well plates to achieve 30% confluence, then infected with the lentivirus (Genechem Co., Ltd, Shanghai) and PCID2 shRNA reagent according to the manufacturer’s instructions. The PCID2 shRNA contains two shRNAs against PCID2 (target region 1,5′-CAC​TGC​ACA​GAG​AGT​AAC​ATA-3’; target region 2,5′-CAC​CTA​CAG​GAA​CCT​CTT​TAA-3′) and negative control shRNA (shCtrl) at an MOI of 15–25. Then puromycin (4 μg/mL, Gibco, America) was added to lentivirus-infected cells to enhance infection efficiency for 2 weeks. Positive cells were selected with puromycin, resulting in establishment of a stable cell line.

### 2.10 Cell counting Kit-8 (CCK-8) assay

CCK-8 assay was used to detect the proliferation of HepG2 and Huh7 cells according to the manufacturer’s protocol. Cells in the logarithmic growth phase were harvested, digested with 0.25% trypsin, and resuspended. Cells (1 × 10^4^ cells/well) were seeded into 96-well plates and then cultivated for 24, 48, and 72 h at 37 °C in a 5% CO_2_ humidified incubator. Subsequently, the culture medium was replaced with MEM complete medium containing different concentrations of β-PGG (3.125, 6.25, 12.5, 25, 50, 100, 200 μmol/L), with six replicates per group. After 24, 48, and 72 h of incubation, 10 μL of CCK-8 reagent was added to each well, and the plates were further incubated for 2–4 h. The absorbance at 450 nm (A450 nm) was measured using a microplate reader (Thermo Fisher, MA, USA) to assess cell viability. The experiment was repeated three times.

### 2.11 5-Ethynyl-2′-deoxyuridine (EdU) proliferation assay

HepG2 and Huh7 cells were digested with 0.25% trypsin and seeded in 96-well plates (5 × 10^3^ cells/well). After cells adhesion, β-PGG (25 μmol/L) was added for 24h. EdU was then added, and the cells were incubated at 37 °C for 2 h. Following fixation, cells proliferation was assessed using the Click-iT EdU Cell Proliferation Kit with Alexa Fluor 594 (Biosharp, Beijing, China) was used to assess HCC cell proliferation according to the manufacturer’s instructions, and the nuclei were counterstained with DAPI. Representative images were captured using a fluorescence microscope (Zeiss LSM900, Jena, Germany) at × 200 magnification with appropriate excitation and emission spectra, and data were analyzed using ImageJ software (NIH, USA). The experiment was repeated three times.

### 2.12 Colony formation assay

The two types of HCC cells, HepG2 and Huh7, were seeded into 6-well plates at a density of 5,000 cells per well. After allowing cells to adhere for 2 days and form initial microcolonies (1-2 clones), the culture medium was replaced on the third day with MEM basal medium containing different treatments. Experimental groups included a control group and three treatment groups with β-PGG at concentrations of 12.5, 25, and 50 μmol/L. After 24 h of treatment, the medium was replaced with drug-free MEM, and half of the medium was refreshed every 5 days. The culture was maintained for 14 days. At the end of the incubation period, the medium was aspirated, and cells were washed once with PBS. Colonies were then fixed with 4% paraformaldehyde for 30 min, followed by staining with crystal violet staining solution for 15 min. After staining, excess dye was removed by rinsing with tap water, and the plates were air-dried. The number of colonies was counted and documented by photography. The experiment was repeated three times, and the number of colony formations was calculated using ImageJ software.

### 2.13 Wound healing assay

HepG2 and Huh7 cells in the logarithmic growth phase were seeded into 6-well plates at a density of 6 × 10^5^ cells per well. After allowing the cells to attach overnight, a sterile 10 μL pipette tip was used to create a linear scratch in the cell monolayer. Detached cells were removed by washing with PBS, followed by the addition of MEM basal medium containing β-PGG at different concentrations (12.5, 25, and 50 μmol/L). The cells were incubated under these conditions for 72 h. The experiment was repeated three times, and the scratch area was quantified using ImageJ software.

### 2.14 Transwell invasion assay

The Transwell invasion assay was conducted using Corning chambers coated with Matrigel. One day prior to the experiment, 24-well plates, Corning chambers, and pipette tips were pre-cooled at −4 °C. The pre-cooled Transwell chambers were then placed into 24-well plates, which were kept on ice. A total of 100 μL of a mixture of serum-free medium and Matrigel (meduim: Matrigel = 8:1) was evenly added into each chamber, followed by incubation in a CO_2_ incubator for 3 h to allow Matrigel polymerization and formation of the basement membrane. After polymerization, the excess liquid in the chambers was removed, and 100 µL of serum-free medium was added into each chamber and incubated for an additional 30 min to hydrate the basement membrane. HepG2 and Huh7 cells were digested with 0.25% trypsin, resuspended, and adjusted to a density of 3 × 10^5^ cells/mL. A total of 100 µL of cell suspension was seeded into the upper chamber. The experiment included a control group and three treatment groups. For the treatment groups, 100 µL of medium containing three different concentrations of β-PGG (12.5, 25, and 50 μmol/L) were added to the upper chamber, while 500 µL of complete medium containing 30% FBS was added to the lower chamber. Cells were cultured at 37 °C for 48 h. After incubation, the chambers were removed, and the medium was aspirated. The inserts were washed three times with PBS, fixed with 500 µL of 4% paraformaldehyde for 30 min, and washed again three times with PBS. The membranes were then stained with 0.1% crystal violet for 15 min, followed by PBS washes (three times). Non-invading cells and residual Matrigel on the upper surface of the membranes were gently wiped off with cotton swabs. After air-drying, the inserts were mounted on slides, and cells that migrated through to the lower surface were observed under an inverted microscope. Five random fields per insert were photographed. The experiment was performed in triplicate, and cell numbers were quantified using ImageJ software.

### 2.15 The Annexin V-FITC/PI dual staining assay

Apoptosis was assessed using flow cytometry with Annexin V-FITC/PI dual staining. HepG2 and Huh7 cells were seeded into 6-well plates (6 × 10^5^ cells/well). The experiment included a control group and three treatment groups. After cell attachment overnight, the treatment groups were exposed to basal medium containing β-PGG at different concentrations (12.5, 25, and 50 μmol/L) for 12 h. After treatment, the culture medium from both the control and treated groups was collected into centrifuge tubes. Cells in each well were then digested with 200 µL of 0.25% trypsin, transferred into the corresponding centrifuge tubes, and combined with the previously collected medium. The cells were resuspended and centrifuged at 500 *g* for 5 min, and the supernatant was discarded. The pellet was gently resuspended in PBS, followed by two additional washes (500 g, 5 min each) to completely remove residual trypsin. The final cell pellets were resuspended in 100 µL of diluted 1× Annexin V binding buffer. Subsequently, 2.5 µL of Annexin V-FITC reagent was added, and the mixture was gently vortexed and incubated in the dark at room temperature for 15–20 min. After incubation, cells were gently resuspended in 200 µL of diluted 1× Annexin V binding buffer, followed by the addition of 2.5 µL of PI reagent. Samples were transferred into flow cytometry tubes and analyzed within 1 h using flow cytometry. The experiment was repeated three times.

### 2.16 Cell cycle analysis by flow cytometry

Cell cycle distribution was analyzed using a NovoCyte Advanteon Dx VBR flow cytometer (Agilent Bio, Hangzhou, China) with propidium iodide (PI) staining. HepG2 and Huh7 cells were seeded into 6-well plates (6 × 10^5^ cells/well). After allowing cell attachment overnight, the cells were treated with three different concentrations (12.5, 25, and 50 μmol/L) of β-PGG for 12 h. Following treatment, adherent cells were detached using 0.25% trypsin (without EDTA) and collected along with the culture medium to ensure inclusion of all cells. The cell suspension was centrifuged at 500 *g* for 5 min, and the supernatant was discarded. Cells were washed once with PBS, resuspended in 300 µL PBS, and centrifuged again at 500× g for 5 min. The supernatant was removed, and cells were resuspended in 300 µL PBS. While gently vortexing, 1.2 mL of ice-cold absolute ethanol (pre-cooled to −20 °C) was slowly added to fix the cells. The suspension was thoroughly mixed and stored at −20 °C overnight. The following day, the fixed cells were centrifuged at 500 *g* for 5 min to remove ethanol, washed once with 1 mL PBS, and incubated at room temperature f or 15 min. After centrifugation at 500 *g* for 5 min, the supernatant was discarded, and the cell pellet was resuspended in 100 µL RNase A solution. The suspension was incubated in a 37 °C water bath for 30 min to degrade RNA. Subsequently, 400 µL of PI solution (50 μg/mL) was added, mixed thoroughly, and incubated at 2 °C–8 °C for 30 min in the dark. Before analysis, the cells were gently resuspended, and the fluorescence intensity of PI was detected by flow cytometry using a 488 nm excitation wavelength. The experiment was repeated three times.

### 2.17 Western blot (WB) analysis

HepG2 and Huh7 cells were treated with three different concentrations (12.5, 25, and 50 μmol/L) of β-PGG for a 24-h period. Following treatment, cells were harvested, and total protein was extracted using RIPA lysis buffer. Protein concentrations were determined using the BCA assay. Loading buffer was added to the samples, followed by denaturation at 95 °C for 5 min. Proteins were separated via SDS-PAGE and transferred onto PVDF membranes. After blocking with a blocking buffer, membranes were incubated overnight at 4 °C with primary antibodies targeting PCID2, CDK6, CyclinD1, *p*-PI3K, PI3K, *p*-Akt, and Akt, all diluted at 1:2000. The following day, membranes were washed with TBST and incubated with species-specific HRP-conjugated secondary antibodies (diluted 1:4,000) at 37 °C for 1 h. After additional washes with TBST, protein bands were visualized using a chemiluminescence detection system. ImageJ software was used to capture images and quantify the grayscale intensity of the protein bands for relative expression analysis. The experiment was repeated three times.

### 2.18 Statistical analysis

All experimental data are reported as the mean ± standard deviation (SD). Statistical comparisons between groups were performed using two-tailed Student’s t-test for unpaired samples. *P* value <0.05 was considered statistically significant.

## 3 Results

### 3.1 Prediction and validation of the PCID2 active sites

Structure-based virtual screening (VS) via molecular docking is a powerful approach for identifying potential inhibitors by predicting binding conformations and assessing docking scores. Accurate identification of the target protein’s active sites is a critical for successful docking studies. To date, no crystal structures of PCID2 in complex with its inhibitors have been reported. Therefore, SiteMap was employed to predict potential active sites on PCID2, yielding five candidate binding pockets (Supplementary Table S1). Among these, site_1 and site_2 satisfied all three key evaluation criteria: SiteScore >0.8, Dscore >0.8, and binding pocket volume >100 Å^3^.

Previous studies have shown that the intrinsically disordered protein DSS1 (26S proteasome complex subunit) can modulate the PCID2 conformation and enhance its structural stability upon binding (Ellisdon and Stewart, 2012; Kim et al., 2011). Comparative analysis indicated that site_1 is located at the interface of the superhelical domain, the winged helix (WH) domain of PCID2, and DSS1, comprising residues Pro266, Thr267, Val268, Glu269, Leu271, Ala280, Thr283, Arg284, Ser287, Glu288, Arg323, Asn324, Lys327, Lys328, and Leu331 (PCID2), and Glu40, Asn42, Trp43, Asp44, Asp45, Asn47, Val48, Glu49, and Asp50 (DSS1). In contrast, site_2 residues solely within the superhelical domain, distal to the WH domain and DSS1. Consequently, site_1 was selected for subsequent docking and functional analyses.

To validate site_1 and assess the docking reliability, FO (N-*trans*-feruloyloctopamine), a previously identified PCID2 inhibitor (Bai et al., 2017), was selected as a reference molecule and docked into site_1. The docking score was −6.533 kcal/mol. The docked conformation and interaction diagram of the FO-PCID2 complex are shown in Supplementary Figures S1A-C. Interaction analysis revealed hydrogen bonds between FO and with Val268/Ala280 (PCID2), and Glu40′Asp50 (DSS1), stabilizing FO within the binding pocket. Notably, FO’s backbone -OH simultaneously interact with Val268 (PCID2) and Glu40 (DSS1), likely enhancing DSS1 binding and stabilizing PCID2. Hydrophobic and electrostatic interactions further contributed to binding stability.

MD simulations of both apo-PCID2 and the FO-PCID2 complex showed stable RMSD (root mean square deviation) and radius of gyration (R_g_) profiles (Supplementary Figures S1D-F), suggesting system equilibrium; notably, the FO-PCID2 complex exhibited a more compact structure. Root mean square fluctuation (RMSF) analysis of C_α_ atoms in the FO-PCID2 complex (Supplementary Figures S1G) revealed structural stability of PCID2, while the WH domain (residues 309–399, highlighted in yellow) exhibited greater flexibility.

Binding free energy analysis using the MM-GBSA method yielded a ΔG_*bind*_ of −14.881 kcal/mol for FO-PCID2., with van der Waals contribution (ΔG_*vdw*_ = −24.232 kcal/mol) and electrostatic contribution (ΔG_*ele*_ = −13.787 kcal/mol), suggesting hydrophobic interactions are the primary driving force for FO binding to PCID2. Per-residue energy decomposition (Supplementary Figures S1H) identified key contributors (ΔG >1.0 kcal/mol), including Met61/Pro62 (PCID2) (corresponding to Met265/Pro266 in the PDB file), and Glu198/Trp201 (DSS1) (corresponding to Glu40/Trp43), consistent with the docking results.

### 3.2 Discovery of hit compounds and interaction analysis

Following validation of site_1, compounds from the prepared chemical library were docked using a hierarchical strategy (HTVS→SP→XP). Top 10% of compounds were retained after HTVS and SP, while the top 1% was selected post-XP, yielding 339 candidates. To refine the selection, the docking poses of these compounds were manually inspected with particular attention to hydrophobic interactions and critical hydrogen bonding. Compounds with unfavorable binding conformations or bulky substituents protruding beyond the binding pocket were excluded from further consideration.

Eight compounds were shortlisted as potential candidates, based on docking scores, structural diversity and commercial availability (Table 1; Figure 1B). Among these, compound #02 exhibited the highest docking score (−11.044 kcal/mol). As illustrated in Figures 1C–E, compound #02 forms multiple hydrogen bonds with PCID2 (Glu269, Glu288, Ala280, Ser287, Glu288, Lys328) and DSS1 (Asp45, Asn47, Glu49), suggesting that hydrogen bonding plays a crucial role in stabilizing the interaction between #02 and the PCID2-DSS1 complex. For electrostatic interactions, compound #02 interacts with Glu269, Glu281, Arg284, Glu288, and Lys328 of PCID2, as well as with Asp45, Glu49, Asp50, and Asp51 of DSS1. Hydrophobic interactions are also observed between #02 and the PCID2 residues Val268 and Ala280.

**TABLE 1:** Results of molecular docking analysis for the candidate compounds and information.

NO.	Name	CAS.no.	Docking score (kcal/mol)	Source plants	Pharmacological activities	Ref.
#01	Heptasaccharide	121591–98-8	−10.977	Xylooligosaccharides	oxidized cellulose oligosaccharides	Chen et al. (2018), Ye et al. (2011)
#02	1,2,3,4,6-Penta-O-galloyl-β-D-glucose	14937–32-7	−11.044	*Galla Chinensis*, Teaetc.	antioxidant, anti-inflammatory, and antitumor activities	Tong et al. (2021), Zeng et al. (2022)
#03	echinacoside	82854–37-3	−10.87	*Cistanche*	anti-osteoporotic and antitumor activity	Li et al. (2012), Li et al. (2014), Tang et al. (2020)
#04	1,2,3,6-tetragalloylglucose	79886–50-3	−10.272	Arbutin	antimicrobial activity	Park et al. (2018)
#05	Hosenkoside C	156764–83-9	−10.262	Impatiens	—	Li et al. (2011)
#06	1F-Fructofuranosylnystose	59432–60-9	−10.739	Morinda officinalis	Dietary components	Chen and Zhuang (2013)
#07	Stachyose tetrahydrate	10094–58-3	−10.899	*Rehmannia glutinosa*	Hypoglycemic effect	Li et al. (2020)
#08	Chebulinic acid	18942–26-2	−10.647	Terminalia chebula	antitumor activity	Wu et al. (2018)

### 3.3 Effects of candidate compounds on the HCC cell viability

**FIGURE 2:**
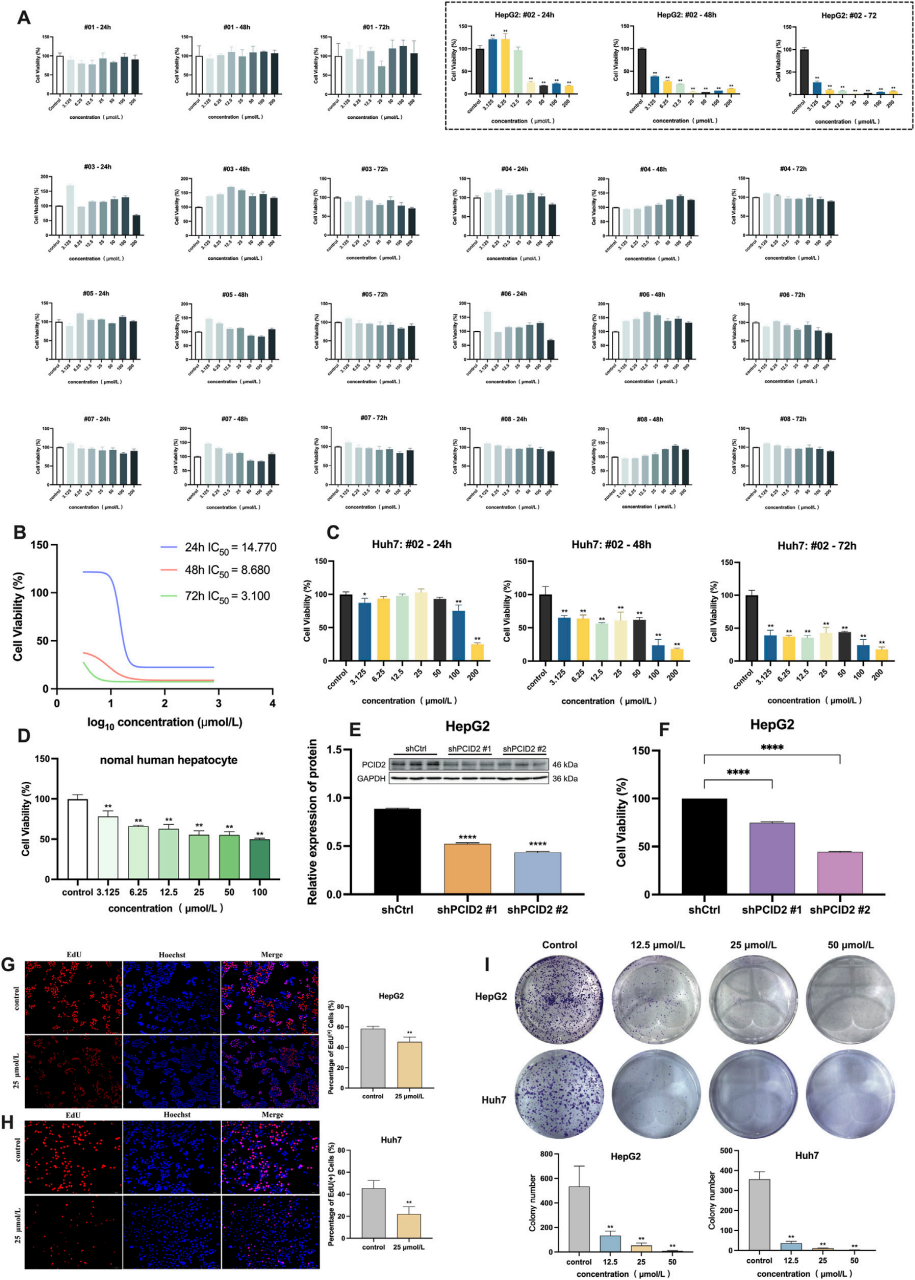
**(A)** Cell viability of HepG2 cells after 24, 48, and 72 h of treatment with eight candidate compounds by CCK-8 assay, and the dashed box displays the results for compound #02 (β-PGG). **(B)** IC_50_ values of the β-PGG on the viability of HepG2 cells. **(C)** Cell viability of Huh7 cells after 24, 48, and 72 h of treatment with β-PGG. **(D)** Cell viability of normal human hepatocytes after 24 h of treatment with β-PGG. **(E)** Validation of PCID2 knockdown efficiency using Western Blot assays. **(F)** Cell viability of PCID2 knockdown in HepG2 cells after 24 h; **(G,H)** Viability and proliferation of the HepG2 **(G)** and Huh7 **(H)** cells after β-PGG treatment by EdU assay (scale bar: 500 μm). **(I)** The colony formation assay was used to evaluate the effects of β-PGG on the clonogenic proliferation capacity of individual HepG2 and Huh7 cells. Data is expressed as mean ± SD (n = 3). *P < 0.05, **P < 0.01, ****P < 0.0001 indicate statistically significant differences compared to the control group.

To rapidly identify compounds with significant inhibitory effects on HCC cells, the CCK-8 assay was initially employed to evaluate the effects of eight candidate compounds on the viability of HepG2 cells in this study. The intervention concentrations of eight compounds were 3.125, 6.25, 12.5, 25, 50, 100, and 200 μmol/L, with incubation times of 24, 48, and 72 h, respectively. As shown in Figure 2A, compound #02 markedly suppressed proliferation in a dose-dependent manner (dashed box), whereas the remaining seven compounds (#01, #03-#08) showed no significant inhibitory effect on HepG2 cell viability. IC_50_ values for compound #02 were 14.770 μmol/L, 8.680 μmol/L, and 3.100 μmol/L at 24, 48, and 72 h, respectively (Figure 2B).

To determine whether compound #02 has an inhibitory effect in other HCC cell lines, we also used CCK-8 assay to further assess its impact on the viability of Huh7 cells. The intervention concentrations and treatment time of compound #02 were the same as those used in the HepG2 experiment. The results were shown in Figure 2C. It can be observed that compound #02 can also significantly reduce the viability of Huh7 cells, which is consistent with the results in the HepG2 cells, and its inhibitory effect markedly increased with the extension of the treatment time.

Based on the above CCK-8 assay results and the calculated IC_50_ values, three concentrations of compound #02 (12.5, 25, and 50 μmol/L) were set for subsequent experiments. These interventions were further validated in both HepG2 and Huh7 cells.

### 3.4 Validation of β-PGG binding affinity to PCID2

Following VS and CCK-8 assays evaluating its inhibitory effects on HCC cell viability, SPR analysis was subsequently performed to resolve the binding kinetics and molecular affinities between β-PGG and PCID2. Purified PCID2 protein was immobilized on a CM5 sensor chip, with a total coupling density of 5250 RU, confirming successful protein coupling (Supplementary Figure S2). β-PGG was injected in a concentration-gradient manner for kinetic fitting, and the results are shown in Figure 1F. The SPR sensorgrams revealed a direct and high-affinity interaction between β-PGG and human PCID2, with dissociation constant (K_D_) of 5.27 × 10^−6^ M, association rate constant (K_a_) of 9.58 × 10^4^ M^-1^ and dissociation rate constant (K_d_) of 0.505 s^-1^.

Although the SPR data provide compelling evidence supporting β-PGG as a direct binding molecule, of PCID2, it is necessary to incorporate orthogonal validation approaches such as the cellular thermal shift assay (CETSA) and drug affinity responsive target stability (DARTS) for further verification under physiologically relevant conditions, thereby enabling a more comprehensive biophysical characterization of the β-PGG–PCID2 interaction mechanism.

### 3.5 Effects of β-PGG on normal hepatocytes

The potential cytotoxicity of β-PGG on normal hepatocytes was evaluated using the CCK-8 assay after 24 h treatment. As shown in Figure 2D, the results indicated that, in comparison with the control group, β-PGG had a certain inhibitory effect on the viability of normal hepatocytes (*P* < 0.01). However, within the tested concentration range (0–100 μmol/L), the inhibition rate of β-PGG on cells does not exceed 50%, suggesting that the IC_50_ value of its intervention on normal cells is greater than 100 μmol/L. These results indicate that under the experimental conditions of this study, β-PGG exhibits relatively low cytotoxicity towards normal hepatocytes.

### 3.6 β-PGG inhibits HCC cell proliferation

To investigate the functional role of PCID2 in HCC, HepG2 cell lines with stable PCID2 knockdown were established. Western Blot analysis confirmed a marked reduction in PCID2 protein expression, among which shPCID2#1 and shPCID2#2 demonstrated greater silencing efficiency compared with shCtrl (*P* < 0.001; Figure 2E). Therefore, these two constructs were selected for subsequent experiments. CCK-8 assay results showed that PCID2 knockdown significantly suppressed the proliferative capacity of HepG2 cells (*P* < 0.001; Figure 2F).

We next evaluated the anti-proliferative effect of β-PGG in HCC cells. In addition to the CCK-8 assay described in Section 3.3, an EdU incorporation assay was conducted to further assess its impact on the proliferation rates of HepG2 and Huh7 cells. As shown in Figures 2G,H, β-PGG treatment resulted in a significant reduction in the proportion of EdU + cells compared with the control group (*P* < 0.01), indicating that β-PGG suppresses DNA synthesis and thereby inhibits HCC cell proliferation.

The colony formation assay was performed to assess the inhibitory effect of β-PGG on the long-term proliferative capacity of HepG2 and Huh7 cells. HepG2 and Huh7 cells were treated with β-PGG at concentrations of 12.5, 25, and 50 μmol/L for 24 h, followed by an additional 2-week culture period to allow colony formation. As shown above in Figure 2I, β-PGG treatment significantly reduced the colony formation rate of both cell lines compared to the control group (*P* < 0.01). Moreover, the number of colonies decreased in a concentration-dependent manner, with higher β-PGG concentrations leading to a greater inhibitory effect (*P* < 0.01). These findings indicate that β-PGG effectively suppresses the long-term proliferative capacity of HepG2 and Huh7 cells.

### 3.7 β-PGG suppresses invasion and migration of HCC cells

**FIGURE 3:**
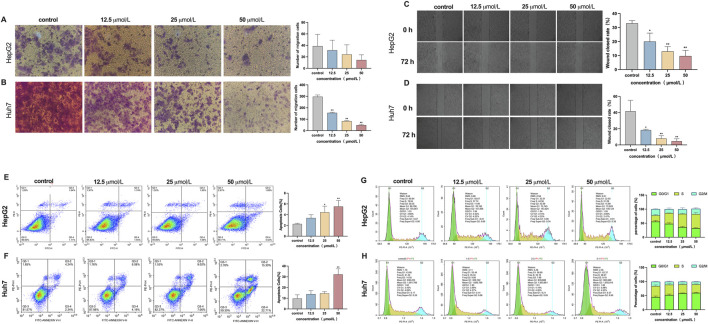
Effect of β-PGG on the malignant phenotype in HepG2 and Huh7 cells, respectively. **(A,B)** The Transwell invasion assay was employed to assess the effects of β-PGG on the invasion ability of HepG2 **(A)** and Huh7 **(B)** cells. **(C,D)** The wound healing assay was conducted to evaluate the effects of β-PGG on the migration ability of HepG2 **(C)** and Huh7 **(D)** cells. **(E,F)** The Annexin V-FITC/PI dual staining assay was employed to evaluate the effects of β-PGG on the apoptosis rate of HepG2 **(E)** and Huh7 **(F)** cells. **(G,H)** Flow cytometry was used to investigate the effects of β-PGG on the cell cycle of HepG2 **(G)** and Huh7 **(H)** cells. Data is expressed as mean ± SD (n = 3). *P < 0.05, **P < 0.01 indicate statistically significant differences compared to the control group.

The Transwell invasion assay was employed to evaluate the effect of β-PGG on the invasive capacity of HepG2 and Huh7 cells. As shown in Figure 3A, following 72-h exposure to β-PGG at concentrations of 12.5, 25, and 50 μmol/L, a reduction in the number of HepG2 cells that migrated through the Transwell membrane was observed compared to the control group. However, no statistically significant difference was observed between the experimental groups and the control group. In contrast, for the Huh7 cell line, the results of the invasion assay (Figure 3B) demonstrated a significantly decreased in the number of cells passing through the Transwell membrane as the concentrations of β-PGG increased. This reduction was statistically significant (*P* < 0.01). These results indicate that β-PGG treatment significantly inhibited the invasive ability of Huh7 cells, while in HepG2 cells a similar but statistically non-significant trend was observed, suggesting a more pronounced inhibitory effect in Huh7 cells.

The wound healing assay was conducted to evaluate the effect of β-PGG on the migratory ability of HepG2 and Huh7 cells. Following treatment with 12.5, 25, and 50 μmol/L β-PGG, the scratch closure rates in HepG2 cells were 20.00% ± 6.4%, 13.00% ± 3.7%, and 10.00% ± 3.7%, respectively. These rates were significantly lower than those in the control group, demonstrating reductions of 33% and 10%, respectively (*P* < 0.05). After 72 h of β-PGG treatment, the wound closure rate in all treated groups was markedly lower than that of the control group (Figure 3C). Similarly, for Huh7 cells, the scratch healing rates at β-PGG concentrations of 12.5, 25, and 50 μmol/L were 18% ± 1.1%, 8% ± 3.1%, and 4% ± 2.9%, respectively. These values were significantly lower than the control group (42% ± 13.4%), with statistically significant observed (*P* < 0.05). After 72 h of β-PGG exposure, the wound healing rate in all treated groups remained significantly reduced compared to the control group (Figure 3D). These results suggest that β-PGG effectively inhibits the migratory ability of HepG2 and Huh7 cells in a dose-dependent manner.

### 3.8 β-PGG induces apoptosis and cell cycle arrest

The Annexin V-FITC/PI dual staining assay was employed to evaluate the effect of β-PGG on apoptosis in HepG2 and Huh7 liver cancer cells. As demonstrated in the flow cytometry scatter plots (Figures 3E,F), four distinct cell populations were identified: live cells, early apoptotic cells, late apoptotic cells, and necrotic cells. In HepG2 cells, treatment with β-PGG at concentrations of 12.5, 25, and 50 μmol/L for 24 h led to a significant increase in apoptosis rates compared to the control group (*P* < 0.01), with the highest concentration inducing the most substantial effect (Figure 3E). This pro-apoptotic effect was found to be concentration dependent. For the Huh7 cells, a significant increase in apoptosis was observed at the 50 μmol/L concentration compared to the control group (*P* < 0.01), while the apoptosis rate in the lower concentration groups (12.5 and 25 μmol/L) showed an upward trend, though this difference was not statistically significant (Figure 3F). These results suggest that β-PGG effectively enhances the apoptosis rate of liver cancer cells, demonstrating its pro-apoptotic effect on both HepG2 and Huh7 cells.

Flow cytometry was used to investigate the effects of β-PGG on the cell cycle of HepG2 and Huh7 cells after a 24-h treatment period. The control group was compared with β-PGG treated. In HepG2 cells, β-PGG treatment led to a dose-dependent decrease in the proportion of cells in the G0/G1 phase (from 55.56% to 31.67%) and an increase in the proportion of cells in the S phase (from 22.43% to 50.46%) (Figure 3G), compared to the control group. These results indicate that β-PGG significantly enhanced the percentage of HepG2 cells in the S phase with a statistically significant difference (*P* < 0.001). For Huh7 cells, β-PGG treatment resulted in an increase in the proportion of cells in the G0/G1 phase (from 44.92% to 60.01%) and a decrease in the proportion of cells in the S phase (from 39.28% to 30.06%) with increasing concentrations (Figure 3H). These results suggest that β-PGG significantly increased the percentage of Huh7 cells in the G0/G1 phase compared to the control group (*P* < 0.001), with a statistically significant difference. The findings from this study demonstrate that the effect of β-PGG on the cell cycle are cell-type-specific. Specifically, β-PGG-induced cell cycle arrest in the S phase in HepG2 cells, while it induced cell cycle arrest in the G0/G1 phase in Huh7 cells. These findings suggest that β-PGG can induce cell cycle arrest in either the G0/G1 phase or the S phase, depending on the liver cancer cell line.

### 3.9 Effect of β-PGG on cell cycle proteins and PI3K/Akt signaling

#### 3.9.1 Effect of β-PGG on PCID2 protein expression

**FIGURE 4:**
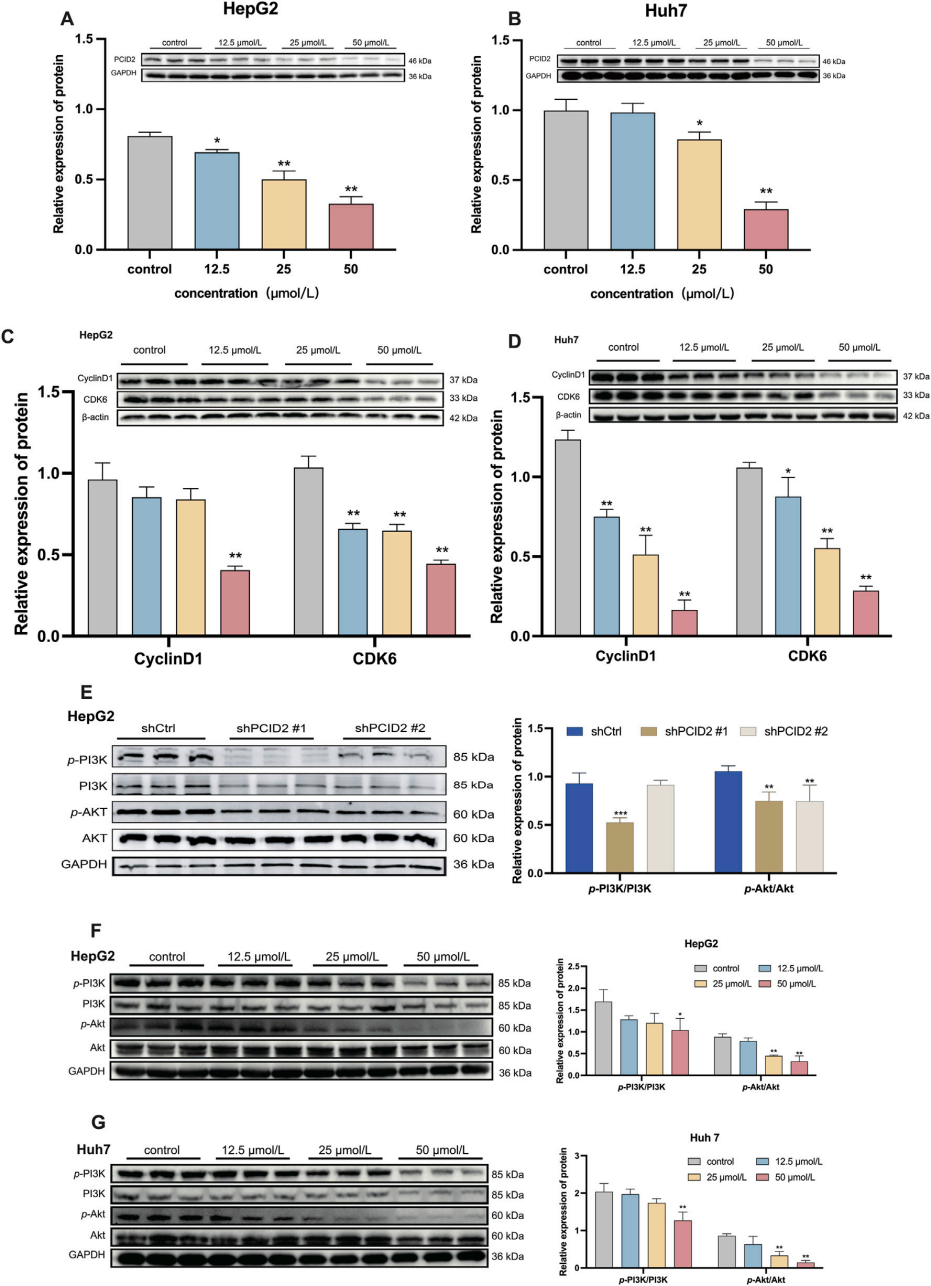
Western blot analysis of protein expression levels. **(A,B)** PCID2 expression in HepG2 **(A)** and Huh7 **(B)** cells. **(C,D)** Expression of the cell cycle-related proteins CyclinD1 and CDK6 in HepG2 **(C)** and Huh7 **(D)** cells following β-PGG treatment. **(E)** Expression levels of *p*-PI3K/PI3K and *p*-Akt/Akt in PCID2 knockdown HepG2 cells. **(F,G)** Expression levels of *p*-PI3K/PI3K and *p*-Akt/Akt in HepG2 **(F)** and Huh7 **(G)** cells following β-PGG treatment. Data is expressed as mena ±SD (n = 3). *P < 0.05, **P < 0.01, ***P < 0.001, and ****P < 0.0001 indicate statistically significant differences compared to the control group.

To further validate the inhibitory effect of β-PGG on PCID2, we assessed the protein expression levels of PCID2 in HepG2 and Huh7 cells following β-PGG treatment. HepG2 cells were treated with β-PGG at concentrations of 12.5, 25, and 50 μmol/L for 24 h. The results showed a significant, dose-dependent reduction in PCID2 protein expression compared to the control group (*P* < 0.05; Figure 4A). A similar trend was observed in Huh7 cells, with β-PGG treatment also leading to a marked decrease in PCID2 expression (Figure 4B). These findings indicate that the intervention of β-PGG could effectively reduce PCID2 protein expression *in vitro,* supporting a potential mechanistic link between β-PGG and PCID2 suppression.

#### 3.9.2 β-PGG suppresses the expression of cell cycle-related proteins

Considering that cell-cycle dysregulation and apoptosis resistance are hallmarks of tumorigenesis, we investigated the effects of β-PGG on key regulators of cell cycle progression in HepG2 and Huh7 cells. As shown above in Figures 3G,H (Results 3.8), β-PGG impedes the progression of HCC cells from the G0/G1 or S phase to the G2/M phase. To further elucidate the underlying mechanism, we examined the expression of key regulatory proteins involved in the G0/G1 transition, including Cyclin D1 and CDK6. HepG2 cells were treated with β-PGG at concentrations of 12.5, 25, and 50 μmol/L for 24 h. WB analysis revealed that the expression levels of Cyclin D1 and CDK6 were significantly downregulated in a dose-dependent manner compared to the control group (*P* < 0.01; Figure 4C). Similarly, in Huh7 cells, β-PGG treatment for 24 h also markedly reduced the expression of Cyclin D1 and CDK6 compared to untreated controls (*P* < 0.05; Figure 4D). These findings collectively indicate that β-PGG downregulates the expression of Cyclin D1 and CDK6 in both HepG2 and Huh7 cells, suggesting its inhibitory effect on cell cycle progression at the G0/G1 phase.

#### 3.9.3 β-PGG inhibits phosphorylation of PI3K and Akt in HCC cells

Based on our previous findings (Bai et al., 2017; Bai et al., 2022), we hypothesized that PCID2 may promote HCC progression through activation of the PI3K/Akt signalling pathway. To examine this possibility, we first evaluated the expression of total and phosphorylated PI3K and Akt in PCID2-knockdown cells. As shown in Figure 4E, the levels of phosphorylated Akt (*p*-Akt) and PI3K (*p*-PI3K) were significantly reduced in PCID2-silenced cells (shPCID2#1) compared with shCtrl group (*P* < 0.01), suggesting that PCID2 may enhance the proliferation and invasion phenotypes of HCC cells by activating the PI3K/Akt pathway.

We then investigated whether β-PGG could modulates this pathway. In HepG2 cells, β-PGG treatment resulted in a dose-dependent decrease in the levels of *p*-PI3K and *p*-Akt; however, statistical significance was observed only in the high-dose group compared with the control group (*P* < 0.01; Figure 4F). Consistent results were obtained in Huh7 cells (*P* < 0.01; Figure 4G). We further assessed the expression of total PI3K and Akt proteins and found that total Akt expression remained unchanged compared with the control group, whereas a significant decrease in total PI3K levels was detected in the high-dose β-PGG group. This trend was consistent with the changes observed on PCID2 knockdown. These findings suggest that additional mechanisms beyond direct pathway inhibition may be involved.

These above results indicate that the effects of β-PGG treatment on the PI3K/Akt pathway were consistent with those observed in PCID2-knockdown cells, with both interventions significantly suppressing *p*-PI3K and *p*-Akt expression. This similarity suggests that β-PGG inhibits activation of the PI3K/Akt signaling pathway in a manner that resembles PCID2 silencing, implying that the anti-HCC effects of β-PGG may be mediated, at least in part, through targeting the PCID2-PI3K/Akt axis.

Collectively, these data demonstrated that β-PGG inhibits the activation of the PI3K/Akt signaling pathway in both HepG2 and Huh7 cells, accompanied by a pronounced decrease in Cyclin D1, CDK6, and PCID2 expression. These findings support that β-PGG may exert its anti-HCC effects by suppressing PI3K/Akt pathway activation and disrupting cell-cycle progression.

## 4 Discussion

HCC remains a highly aggressive malignancy characterized by rapid progression, high recurrence rates, and poor prognosis (Storandt et al., 2022). Early-stage HCC is often asymptomatic, and most patients are diagnosed at advanced stages when curative surgery is no longer feasible. Consequently, stage-dependent therapies are adopted, with systemic antitumor agents constituting the cornerstone of treatment for intermediate and advanced HCC (Yang et al., 2023). Sorafenib, a multi-kinase inhibitor targeting vascular endothelial growth factor, was the first molecular targeted drug approved for systemic HCC therapy and remains the standard first-line treatment for patients with unresectable or advanced disease (Llovet et al., 2008; Tang et al., 2020).

β-PGG, a polyphenolic gallotannin predominantly derived from *Rhus chinensis* (five-leafed Galla) and also present in tea (*Camellia sinensis*), hawthorn (*Crataegus pinnatifida*), and grapes (*Vitis vinifera*), has been reported to exhibit diverse pharmacological activities, including antitumor, antioxidant, anti-inflammatory, and antidiabetic effects (Jie et al., 2024; Lin et al., 2011; Wang and Ho, 2009; Zhang et al., 2009). In the present study, we demonstrate that β-PGG exerts potent anti-tumor effects in HCC cells by significantly suppressing proliferation, impairing migratory and invasive capacities, inducing apoptosis, and causing cell cycle arrest. It is noted that the inhibitory effect on invasion was statistically significant in Huh7 cells, whereas in HepG2 cells a similar but non-significant trend was observed, suggesting potential cell line-specific differences in sensitivity to β-PGG. Notably, β-PGG showed lower cytotoxicity toward normal hepatocytes than toward HepG2 cells, which was not consistently reproduced in Huh7 cells. This discrepancy suggests that the cytotoxic selectivity of β-PGG is not consistent across different HCC cell types, raising concerns about its potential therapeutic window. Several factors may contribute to the observed difference. HepG2 and Huh7 cells have distinct genetic and metabolic backgrounds, which could influence drug uptake, detoxification capacity, and susceptibility to cell death pathways. Importantly, the current observations are limited to a small panel of cell types and thus do not provide definitive evidence regarding the safety profile of β-PGG. This constitutes one of the limitations of the present study. These findings highlight the need for deeper evaluation of β-PGG safety and specificity, as well as structural optimization if it is to be further developed as a lead compound. Further investigations, such as applying additional cell lines and complementary assays, will provide more robust evidence to support the potential therapeutic application of β-PGG. Taken together, these considerations emphasize that a comprehensive and systematic evaluation of the safety of β-PGG is essential and constitutes one of the main directions of our ongoing research.

PCID2, a PCI domain-containing subunit of the TREX2 complex, is known to participate in mRNA export, cell cycle regulation (Cunningham et al., 2014; Vdovina et al., 2023). Loss of PCID2 in B cells disrupts mitotic checkpoint regulation and leads to abnormal cell cycle progression, emphasizing its essential role in mitosis and maintenance of cellular homeostasis (Nakaya et al., 2010). In addition, PCID2 is indispensable for maintaining the self-renewal capacity in both mouse and human embryonic stem cells (ESCs) by preventing differentiation (Ye et al., 2014). These reported findings indicate that PCID2 participates in diverse biological processes, including proliferation, apoptosis, stress response, and stemness, thereby contributing to tumor cell reprogramming and oncogenesis. Despite its emerging significance, the precise molecular mechanisms by which PCID2 promotes HCC progression remain incompletely defined. Mechanistically, our study demonstrates that β-PGG treatment significantly reduced PCID2 expression in HCC cells, accompanied by downregulation of Cyclin D1 and CDK6, two key regulators of the G1-to-S phase transition. This molecular effect is consistent with the observed cell cycle arrest at the G0/G1 or S phases and the effective blockade of G2/M progression. Moreover, our results revealed that β-PGG markedly decreased the phosphorylation levels of PI3K and Akt, indicating suppression the activation of this pathway. The PI3K/Akt pathway is known to regulate Cyclin D1 and CDK6 through multiple mechanisms, promoting cell cycle progression and accelerating the G1 to S phase transition. The noteworthy observation is that β-PGG not only reduced PI3K/Akt phosphorylation levels but also decreased total PI3K protein levels at higher concentrations. This suggests that additional regulatory mechanisms may contribute, including transcriptional repression, enhanced protein degradation, or stress-related processes. Although our current results demonstrate a correlation between PCID2 suppression and PI3K/Akt pathway inhibition, we cannot fully exclude the possibility that β-PGG exerts its effects through direct interaction or alternative targets. Future studies aim to clarify whether β-PGG modulates PI3K expression through transcriptional suppression, enhanced protein turnover, or indirect feedback mechanisms related to apoptosis or proteostasis.

Overall, several limitations should be acknowledged. First, the current conclusions are derived from *in vitro* experiments using a limited number of HCC cell lines, and *in vivo* studies are required to substantiate the therapeutic relevance of the findings. Second, although our data reveal a clear association between PCID2 suppression and pathway inhibition, causal relationships remain to be rigorously established through gain- and loss-of-function rescue experiments. Third, it is not yet clear whether β-PGG directly targets PCID2 or regulates its stability through upstream regulators, and this question should be addressed using approaches such as CETSA and/or DARTS, as well as pulldown-based target profiling. Finally, the selectivity and potential off-target effects of β-PGG must be systematically assessed before considering translational advancement. These limitations contribute to the current mechanistic uncertainties. To address them, ongoing studies in our laboratory, including lentiviral gain- and loss-of-function models, as well as transcriptomic and proteomic analyses, are expected to provide causal evidence for the PCID2-dependent effects of β-PGG and further clarify its biological function role in HCC.

In summary, this study identifies β-PGG as a potential PCID2 inhibitor with notable anti-HCC effects at the cellular level. β-PGG suppresses cell proliferation and migration/invasion, arrests the cell cycle, promotes apoptosis, and attenuates PI3K/Akt signaling, with effects partly dependent on PCID2. Given the urgent need for effective targeted therapies in advanced HCC, PCID2 emerges as a promising therapeutic target. Rational design of selective and low-toxicity small-molecule PCID2 inhibitors may represent a novel strategy for HCC treatment. Importantly, ongoing mechanistic studies and *in vivo* evaluation are warranted to fully elucidate the functional role of PCID2 in HCC pathogenesis and to substantiate the translational potential of β-PGG.

## Data Availability

The raw data supporting the conclusions of this article will be made available by the authors, without undue reservation.

## Supplementary material

SUPPLEMENTARY FIGURE S1 The molecular docking results of FO with PCID2 (PDB code: 3T5X). **(A)** Surface representation of the FO docking conformation at the site_1 active pocket of PCID2. **(B,C)** Binding mode of FO in active sites, and residues within 3 Å and 2D-interaction was shown. D-H show that structural characteristics of apo-PCID2 and FO-PCID2 comples during the MD simulation process: **(D)** RMSD of backbone C_α_ atoms relative to the initial structure over simulation time; **(E)** time-dependent changes of the average radius of gyration (R_g_) for all trajectories in the apo-PCID2 system; **(F)** time-dependent changes of R_g_ for all trajectories in the FO-PCID2 system; **(G)** RMSF of backbone C_α_ atoms per-residue in the FO-PCID2 system; **(H)** binding free energy decomposition of FO-PCID2 system by MM/GBSA method.

SUPPLEMENTARY FIGURE S2 Conjugation diagram of human PCID2 protein by SRP.
