# Supplementary material for: A natural PCID2-Targeting compound suppresses hepatocellular carcinoma progression: evidence from structure-based discovery and biological evaluation
Source: Front Pharmacol. 2025 Nov 28;16:1687517. doi: 10.3389/fphar.2025.1687517 (PMC12698594; doi:10.3389/fphar.2025.1687517)
Supplement: Supplementary file 1 [file Supplementaryfile1.docx]

***Supplementary Material***

**Supplementary Table S1** Predicted active sites of PCID2 identified by SiteMap analysis

| **site ID** | **schematic diagram** | **SiteScore** | **Dscore** | **volume (Å^3^)** |
| --- | --- | --- | --- | --- |
| site_1 | 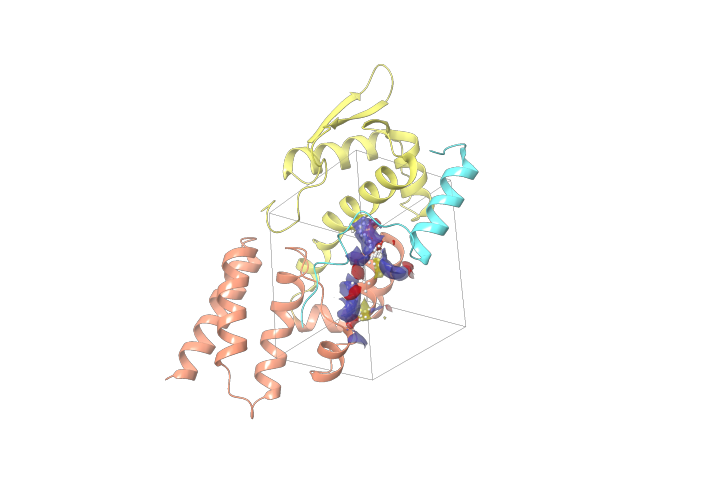 | 0.974 | 1 | 230.496 |
| site_2 | 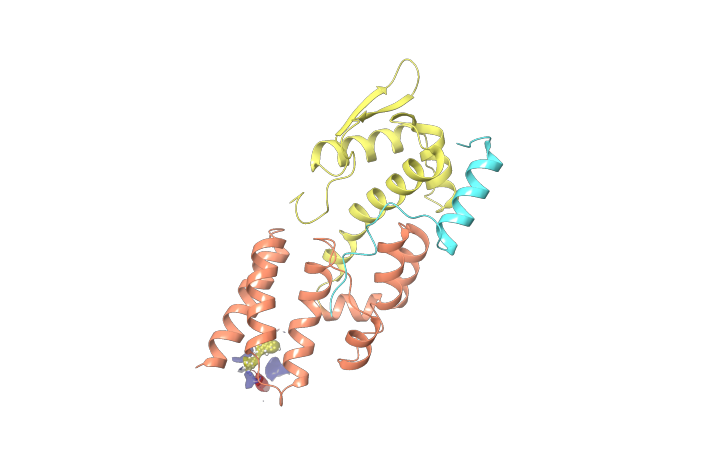 | 0.844 | 0.848 | 140.973 |
| site_3 | 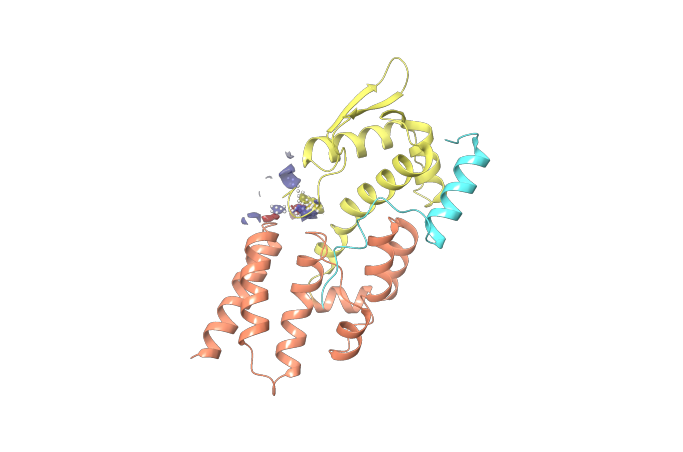 | 0.722 | 0.701 | 117.306 |
| site_4 | 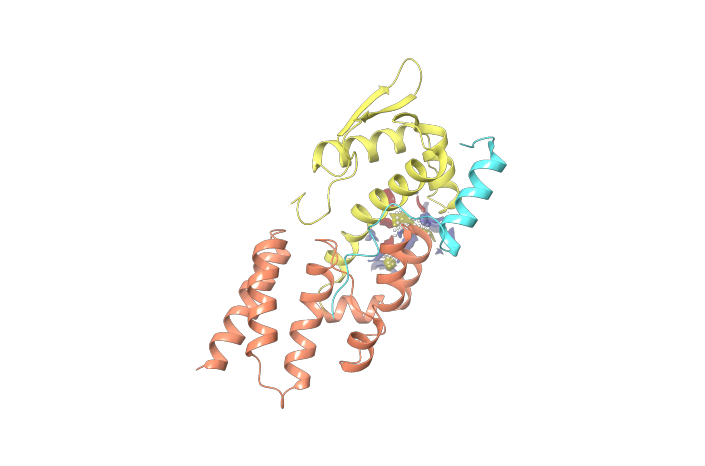 | 0.752 | 0.748 | 129.654 |
| site_5 | 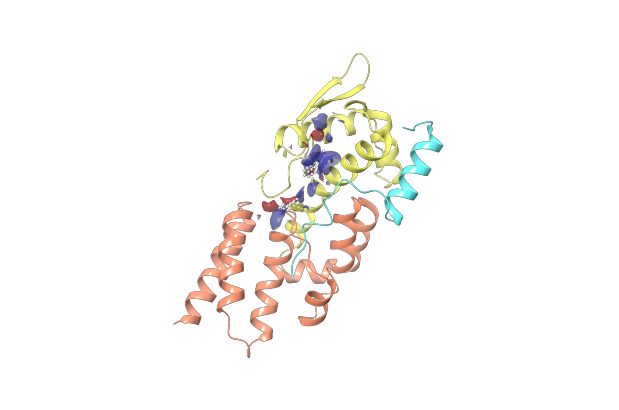 | 0.849 | 0.901 | 85.407 |

The PCID2-DSS1 protein structure is depicted in a ribbon representation, with the WH domain of PCID2 shown in yellow, the superhelical domain of PCID2 in orange, and the DSS1 structure in cyan. The active sites predicted by SiteMap are color-coded as follows: hydrogen bond donor regions in blue, hydrogen bond acceptor regions in red, and hydrophobic regions in yellow.

**Supplementary Figures Legend**

**Supplementary FIGURE S1** The molecular docking results of FO with PCID2 (PDB code: 3T5X). (A) Surface representation of the FO docking conformation at the site_1 active pocket of PCID2. (B, C) Binding mode of FO in active sites, and residues within 3 Å and 2D-interaction was shown. D-H show that structural characteristics of apo-PCID2 and FO-PCID2 comples during the MD simulation process: (D) RMSD of backbone C_α_ atoms relative to the initial structure over simulation time; (E) time-dependent changes of the average radius of gyration (R_g_) for all trajectories in the apo-PCID2 system; (F) time-dependent changes of R_g_ for all trajectories in the FO-PCID2 system; (G) RMSF of backbone C_α_ atoms per-residue in the FO-PCID2 system; (H) binding free energy decomposition of FO-PCID2 system by MM/GBSA method.

**Supplementary FIGURE S2** Conjugation diagram of human PCID2 protein by SRP.
