# Supplementary figures and images for: A natural PCID2-Targeting compound suppresses hepatocellular carcinoma progression: evidence from structure-based discovery and biological evaluation
Source: Front Pharmacol. 2025 Nov 28;16:1687517. doi: 10.3389/fphar.2025.1687517 (PMC12698594; doi:10.3389/fphar.2025.1687517)

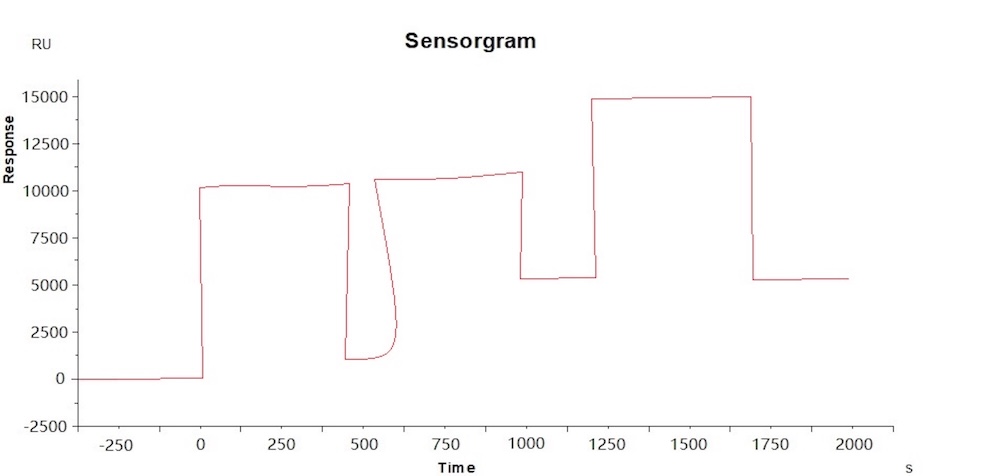

Supplement: Supplementary file 2 [file Image2.jpeg]

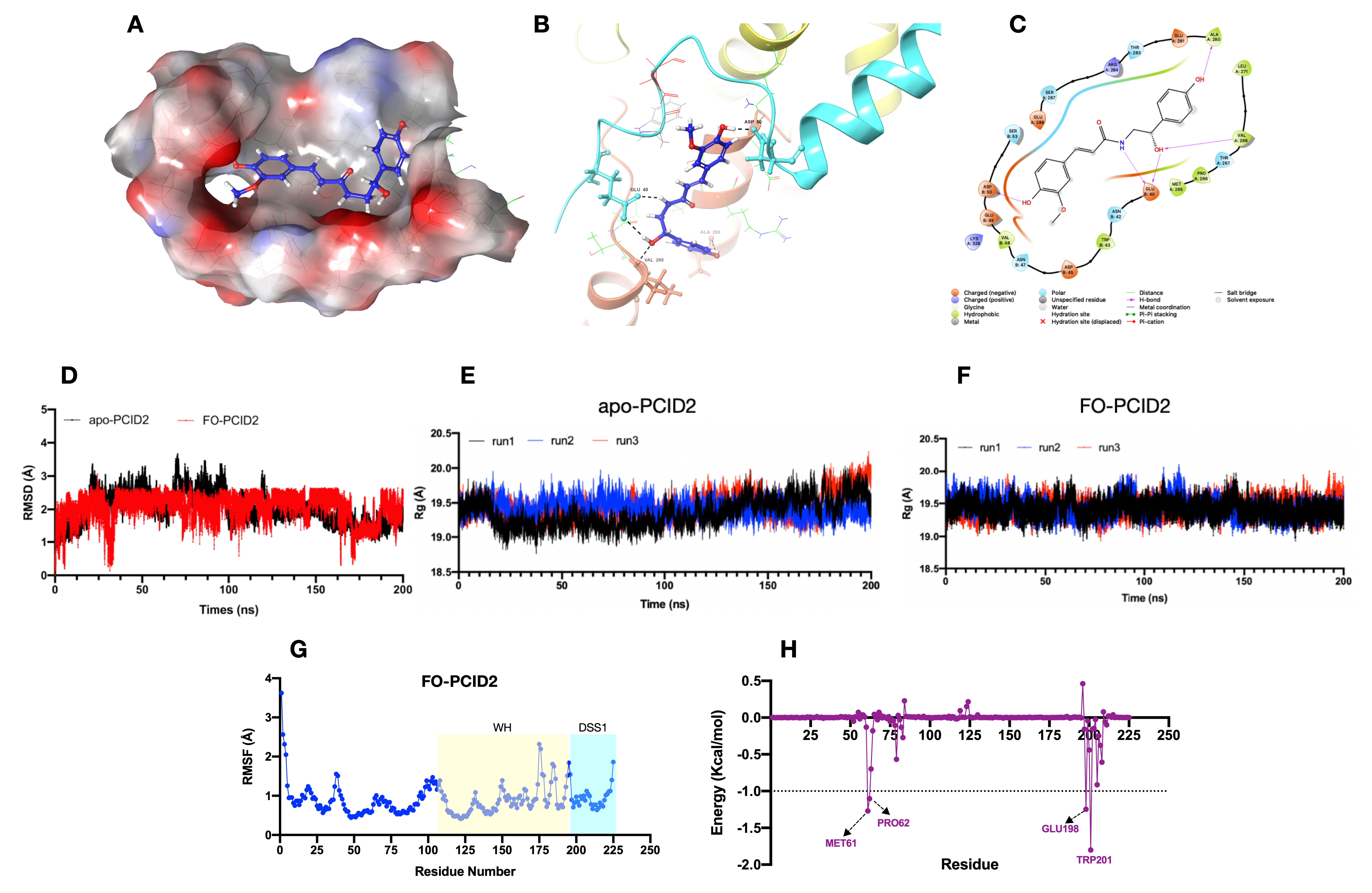

Supplement: Supplementary file 3 [file Image1.png]
